# Critical review of aging clocks and factors that may influence the pace of aging

**DOI:** 10.3389/fragi.2024.1487260

**Published:** 2024-12-13

**Authors:** Mildred Min, Caitlin Egli, Ajay S. Dulai, Raja K. Sivamani

**Affiliations:** ^1^ Integrative Research Institute, Sacramento, CA, United States; ^2^ Integrative Skin Science and Research, Sacramento, CA, United States; ^3^ College of Medicine, California Northstate University, Elk Grove, CA, United States; ^4^ College of Medicine, University of St. George’s, University Centre, West Indies, Grenada; ^5^ Pacific Skin Institute, Sacramento, CA, United States; ^6^ Department of Dermatology, University of California-Davis, Sacramento, CA, United States

**Keywords:** clock, PACE, aging, epigenetic, biological age, microbiome, proteomic

## Abstract

**Background and objectives:**

Aging clocks are computational models designed to measure biological age and aging rate based on age-related markers including epigenetic, proteomic, and immunomic changes, gut and skin microbiota, among others. In this narrative review, we aim to discuss the currently available aging clocks, ranging from epigenetic aging clocks to visual skin aging clocks.

**Methods:**

We performed a literature search on PubMed/MEDLINE databases with keywords including: “aging clock,” “aging,” “biological age,” “chronological age,” “epigenetic,” “proteomic,” “microbiome,” “telomere,” “metabolic,” “inflammation,” “glycomic,” “lifestyle,” “nutrition,” “diet,” “exercise,” “psychosocial,” and “technology.”

**Results:**

Notably, several CpG regions, plasma proteins, inflammatory and immune biomarkers, microbiome shifts, neuroimaging changes, and visual skin aging parameters demonstrated roles in aging and aging clock predictions. Further analysis on the most predictive CpGs and biomarkers is warranted. Limitations of aging clocks include technical noise which may be corrected with additional statistical techniques, and the diversity and applicability of samples utilized.

**Conclusion:**

Aging clocks have significant therapeutic potential to better understand aging and the influence of chronic inflammation and diseases in an expanding older population.

## 1 Introduction

Aging is a multifaceted biological process leading to the gradual decline of physiological functionality and an increased risk of disease and death. In recent decades, the aging population has rapidly increased worldwide, with 11% of the global population being over 60 years old and this statistic expected to rise to 22% by 2050 (Newgard and Sharpless, 2013). A consequence of an aging population is the increased prevalence of chronic diseases, which imposes an economic and psychosocial burden to society. As a result, research related to describing, preventing, and identifying aging processes has expanded rapidly and become increasingly important.

Aging research has delineated the aging process by classifying two separate but interconnected mechanisms: intrinsic and extrinsic aging. Intrinsic aging describes changes in biological hallmarks including cellular and molecular changes, genetics, and hormonal changes that have been described to occur naturally over time (Dodig et al., 2019). Extrinsic aging, however, is regulated by exposure to environmental stressors, dietary habits, oxidative stress, and other factors that accelerate physiologic aging. Traditionally, aging has been quantified by chronological age, which is the exact number of years an individual has lived. However, chronological age does not fully capture the heterogeneity of the aging process, excluding many extrinsic factors that contribute to aging.

Subsequently, the calculation of biological age, which aims to account for interindividual variations in aging rate, has become a topic of interest in aging research. Aging clock models are tools that utilize various modeling approaches to estimate chronological or biological age. Moreover, aging clock models can estimate the rate of aging (ΔAge), otherwise known as the difference between model-predicted biological age and chronological age (Rutledge et al., 2022; Xia et al., 2021). Positive differences between model-predicted biological age and chronological age indicate accelerated aging whereas a negative difference indicates decelerated aging. If the calculated ΔAge exceeds the mean absolute error (MAE) of the aging rate estimation, these individuals can be determined to be fast or slow agers.

An additional marker biological age involves ratios to depict the rate of aging, relative to chronological age. This can be calculated as: (epigenetic age/chronological age) (Borsky et al., 2021). A positive value translates to an increased pace of biological aging, and a negative value depicts a decreased pace of aging. This measure allows for comparability between sample populations which have different average chronological age means.

Aging clocks models may utilize any hallmark changes that occur because of aging, and these may include epigenetic changes, telomere length, genomic stability, altered intercellular communication, chronic inflammation, and gut microbiome dysbiosis, among others (Lopez-Otin et al., 2023). Notably, some of the first aging clock models include the Horvath clock (2013) and Hannum clock (2013), which are both epigenetic clocks modeled after changes in DNA methylation patterns and varying cytosine phosphate guanine (CpG) sites across the genome (Hannum et al., 2013; Horvath, 2013). Several aging clock models have emerged since then, varying from microbiome-based clocks to proteomic clocks (Palmer, 2022).

Recent advancements in the development of large databases, omics technologies, and deep learning models have accelerated the creation of aging clock predictions. Thus, this review aims to summarize the currently available aging clock models, with the goal of identifying existing and potential clinical applications.

## 2 Methods

We performed a narrative review utilizing the following search strategy: PubMed/MEDLINE and Google Scholar databases were searched through August 2024 for articles relevant to this review including a combination of the search terms “aging clock,” “aging,” “biological age,” “chronological age,” “epigenetic,” “proteomic,” “microbiome,” “telomere,” “metabolic,” “inflammation,” “glycomic,” “lifestyle”, “nutrition,” “diet,” “exercise,” “psychosocial,” and “technology.” Articles were reviewed and relevant articles were reviewed for full text and further relevant articles were accessed from their list of references. Articles that adequately described aging clock models, their predictability, methods, and key findings in humans were included. Non-human studies were excluded. Review articles, case reports, case series, abstracts, and communication pieces were excluded.

## 3 Results and discussion

### 3.1 Literature search

After the completion of the initial searches, two independent reviewers performed a preliminary screening assessment and selected full text articles based on relevance. After a final review, 26 articles were extracted based on relevance to the research topic or secondary to scanning the references and data during the full-text review.

### 3.2 Summary of aging clocks

**FIGURE 1:**
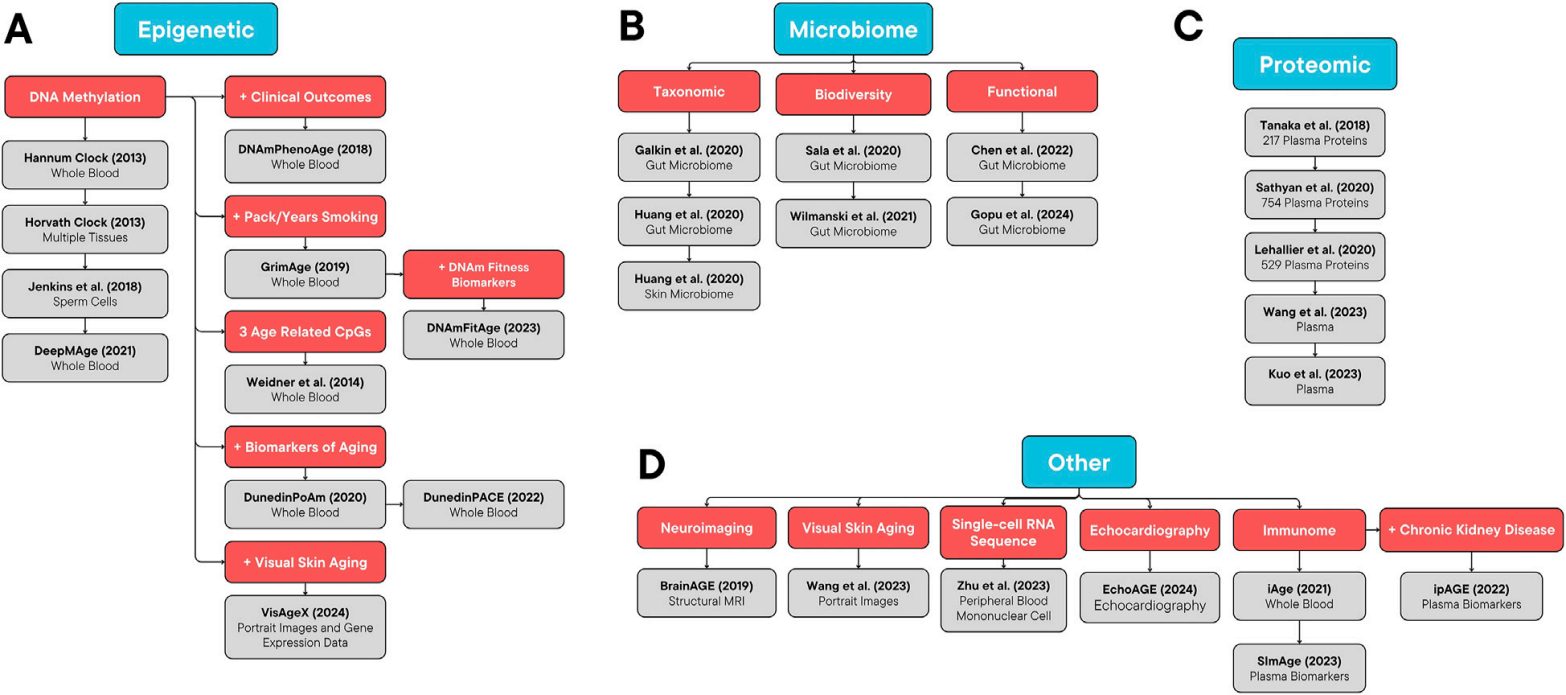
Organizational Framework of Aging Clocks. **(A)** Epigenetic, **(B)** microbiome, **(C)** proteomic, and **(D)** other aging clocks.

Several aging clock models have been developed based on a variety of biological systems that evolve with age. These include clocks based on epigenetic changes, proteomic changes, inflammatory and immune pathways, neuroimaging, microbiome-associated changes, and more (Palmer, 2022). Supplementary Table S1 provides a summary of these clocks. Figure 1 demonstrates an organizational framework of these aging clocks.

#### 3.2.1 Epigenetic clocks

The accumulation of changes in DNA methylation (DNAm) at CpG sites over time has been demonstrated to be related to aging and therefore, several aging clock models based on DNAm patterns have emerged to estimate aging. The Hunnam’s clock was one of the first models based on DNAm, utilizing more than 450,000 CpG markers from whole blood in nearly 700 individuals aged 19 to 101 (Hannum et al., 2013). The predictive accuracy of this methylome-based model was high (R^2^ = 0.963) with an error of 3.9 years. Furthermore, the apparent methylomic aging rate was demonstrated to be faster in men than women, suggesting a sex-related mechanism on rate of aging. Building upon these findings was the Horvath’s clock, which included a wider array of tissues and CpG island patterns (Horvath, 2013). The Horvath’s clock was based on nearly 8000 samples from DNAm array datasets spanning several healthy tissue and cell types with a correlation against chronological age of 0.96 and a MAE of 3.6 years (Horvath, 2013). A notable feature of the Horvath’s clock was its applicability to a variety of tissue and cell types, including whole blood, cerebellum, colon, kidney, liver, and lung, which is a limitation of other aging clock models. The Horvath’s and Hunnam’s clocks are known as first-generation epigenetic aging clocks, and subsequent generations build on their findings to correlate aging with clinical features such as objective laboratory findings and other clinical biomarkers.

The first of these second-generation epigenetic clocks was termed “DNAm PhenoAge” in 2018, a whole-blood-derived epigenetic clock with the intent to incorporate clinical characteristics into a DNAm model utilizing a penalized regression model (Levine et al., 2018). More than 20,000 CpGs were included in the measure, and this model identified novel CpGs that were related to all-cause mortality, aging-related morbidities such as coronary heart disease, smoking, and Alzheimer’s disease. Despite being derived from whole blood samples, the predictability of the DNAm PhenoAge clock in whole blood samples (R^2^ = 0.68) was comparable to that seen across a variety of tissues and cells (R^2^ = 0.71) (Levine et al., 2018). Following this development was the DNAm GrimAge clock, which was designed to predict mortality and has been shown to be strongly predictive of time-to-death (Lu et al., 2019). GrimAge was derived from blood samples of 2,400 individuals and specifically based on 7 DNA methylation-based plasma proteins and pack/years of smoking. The selected plasma proteins were highly correlated with inflammatory processes, cardiovascular diseases, along with kidney and neurological function. Notably, there was a strong association between smoking history and GrimAge (p = 1.7E-47), although there were also correlations with other age-related conditions.

There are a few epigenetic aging clocks that emerged during the creation of these classic epigenetic clocks. In 2014, an epigenetic aging clock based on three specific age-related genes (*ITGA2B*, *ASP*, and *PDE4C*), in which specific CpG islands were analyzed, was developed utilizing whole blood samples (Weidner et al., 2014). Although this model was based on only three genes, it was highly predictive with a mean absolute deviation of 3.34 years, suggesting that aging can be tracked even while using a reduced number of CpGs. In 2018, a DNAm model was built utilizing human sperm from a total of 329 samples, which expanded on the understanding of germ line cells and aging (Jenkins et al., 2018). This model had high predictability (R^2^ = 0.89) and a MAE of 2.04 years. Interestingly, this model was able to predict chronological age with a high degree of accuracy, regardless of fertility status. Nevertheless, more research is needed to establish how external factors may play a role in paternal germ line aging. Of note it these two studies, one utilized mean absolute deviation while the other utilized MAE to describe the data variability. They are not the same measure and the mean absolute deviation gives more insight into the variability in the data while the MAE gives insight into the variability in the error. They are not the same when assessing or comparing different measurement reporting.

In all the cases above, the clocks have been focused on using aging in relation to mortality and the current functionality is not as emphasized. However, later clocks have focused on predictions of the “pace of aging” on a yearly basis. In particular, the DunedinPoAm (Dunedin Pace of Aging Methylation) clock was created with an algorithm to identify the pace of aging to more quantitatively assess for faster or slower aging rates (Belsky et al., 2020). This clock was based on blood samples and tracked biomarkers from 1,037 individuals. The rate of change in each biomarker was compared across all the participants to create a typical rate of change to quantify a pace of aging for each individual study member and to derive an overall pace of aging standard. Interestingly, among individuals with the same chronological age, the individual with the accelerated rate of aging performed more poorly on physical function tests, looked older, and reported worse health. Furthermore, an accelerated rate of aging was associated with lower childhood socioeconomic class and adverse childhood experiences. Notably, this epigenetic clock was not well-correlated with other epigenetic aging clocks, as those were fashioned to estimate age rather than the rate of aging (Belsky et al., 2020). Table 1 summarizes the various measures that were included in the pace of aging calculation. Expanding on this study was the 2022 DunedinPACE (Dunedin Pace of Aging Calculated from the Epigenome), a next-generation blood biomarker for the pace of aging (Belsky et al., 2022). This clock was created using two decades of data, extending past the 12 years of data utilized in the original DunedinPoAm clock. Notably, DunedinPACE incorporated an additional fourth measurement in the fifth decade of life (age 45). As a result of this extended analysis, DunedinPACE provided a more precise pace of aging measurement, with higher test-retest reliability. Interestingly, DunedinPACE had a stronger correlation to chronological age than the DunedinPoAm, which aligns with the age acceleration in later life. Furthermore, DunedinPACE was predictive of morbidity and mortality in older adults and demonstrated increased pace of aging in adults with history of poverty (Belsky et al., 2022). Notably, the DunedinPoAm and DunedinPACE models were derived from longitudinal data collected from a cohort of individuals who were all the same chronological age, thereby avoiding cohort effects typically found in cross-sectional analyses.

**TABLE 1:** Biomarkers contributing to DunedinPoAm and DunedinPACE.

1. Body mass index
2. Waist-hip ratio
3. Glycated hemoglobin
4. Leptin
5. Blood pressure (mean arterial pressure)
6. Cardiorespiratory fitness (V02Max)
7. Forced vital capacity ratio (FEV1/FVC)
8. Forced expiratory volume in one second (FEV1)
9. Total cholesterol
10. Triglycerides
11. High density lipoprotein (HDL)
12. Lipoprotein (a)
13. Apolipoprotein B100/A1 ratio
14. Estimated glomerular filtration rate (eGFR)
15. Blood urea nitrogen (BUN)
16. High Sensitivity C-reactive protein (hs-CRP)
17. White blood cell count
18. Mean periodontal attachment loss (AL)
19. Number of dental-caries-affected tooth surfaces

In 2021, DeepMAge, a DNAm aging clock, was developed using deep learning on nearly 5,000 blood samples to predict age in diseases such as IBD, dementia, and ovarian cancer (Galkin et al., 2021). DeepMAge assigned a higher predicted age to individuals with the listed health conditions, suggesting that these processes were considered in the overall age calculation. Moreover, in 2023, DNAmFitAge was created as a biological age indicator that incorporated levels of physical fitness (McGreevy et al., 2023). This aging clock was based on the DNAmGrimAge clock as mentioned previously and DNAm markers for fitness parameters such as maximal oxygen consumption, forced expiratory volume in one second, handgrip strength, and gait speed. A younger DNAmFitAge was associated with increased levels of fitness and better age-related outcomes, suggesting that improvements in fitness parameters may decelerate the pace of aging (McGreevy et al., 2023).

#### 3.2.2 Telomere length

Telomeres are located at the end of eukaryotic chromosomes and made up of repeats of TTAGGG which bind to proteins to stabilize the telomere. Telomere shortening or damage leads to cellular senescence and contributes to the DNA damage response (d'Adda di Fagagna et al., 2003). Since telomeres shorten each time cells divide, the process can be a biomarker for biological aging (Jylhava et al., 2017). To the best of our knowledge, there are no predictive aging clock models based on telomere length, however, leukocyte telomere length has been shown to shorten at an annual average rate of 30–35 base pairs, reaching about 5–6 kb in individuals over 60 (Calado and Dumitriu, 2013). Centenarians have been shown to maintain telomere length and telomerase activity significantly longer than non-centenarians and thus telomere length is certainly an important component of understanding aging (Vaiserman and Krasnienkov, 2020). One of the challenges in using telomere length is that there are many factors in the variability of telomere length in the repair mechanisms that may make telomere length a less robust predictive tool for biological aging (Vaiserman and Krasnienkov, 2020; Demanelis et al., 2020).

#### 3.2.3 Proteomic clocks

Circulating proteins have been shown to be associated with chronological age and age-related diseases (Kuo et al., 2024). Thus far, two proteomic clocks have been developed to predict chronological age or mortality. One 2018 study characterized the proteomic signature of 1,300 proteins found in the plasma of 240 healthy individuals (Tanaka et al., 2018). Functional pathways that were related to age included proteins that regulated blood coagulation, inflammatory responses, peptidase activity, and cellular apoptosis, among others. After utilizing elastic net regression models, there were 76 key proteins that predicted chronological age accurately. However, there were over 200 proteins that were markedly associated with age (Tanaka et al., 2018). Another study in 2020 performed a similar analysis of nearly 4,300 proteins and found that 754 were robustly associated with chronological age (Sathyan et al., 2020). The most significant proteins associated with chronological age included pleiotrophin, WNT1-inducible-signaling pathway protein 2, chordin-like protein 1, among others. Pleiotrophin, as an example, is involved in cell cycle regulation, cellular migration, and cellular survival as well as acting as a neuromodulator in memory formation. One hundred and sixty-two relevant proteins were carried over to the elastic net regression model, which showed that proteomic age was predictive of chronological age (r = 0.79) (Sathyan et al., 2020).

Similarly, in another study utilizing a proteomic dataset from 3,300 individuals, 491 proteins were found to be highly predictive for aging, with a correlation of 0.98 and MAE of 2.44 during validation testing against chronological age (Lehallier et al., 2020). The six proteins with the most significant change with age included CGA.FSHB, SOST, GDF15, MLN, RET, and PTN. Notably, this proteomic clock demonstrated that individuals that had more experience with aerobic exercise had a younger predicted age than other more sedentary individuals. Furthermore, this study suggested that proteins associated with immune system pathways were especially relevant to predict aging, which is consistent with recent efforts towards generating aging clocks based on the immunome (Lehallier et al., 2020).

There have also been two studies that demonstrate proteomic aging clocks and their relevance in older populations (Kuo et al., 2024; Wang S. et al., 2023). The first study measured 4,712 plasma proteins in nearly 12,000 participants from the Atherosclerosis Risk in Communities (ARIC) study, and estimated age acceleration and its association with chronic diseases and other cofounders (Wang S. et al., 2023). In this study, there were two proteomic aging clocks tested: mid-life and late-life. Between the two proteomic clocks, there were four common proteins: PTN, ADAMTS-5, MMP12, and CDON, several of which are also common to previously mentioned proteomic clocks (Wang S. et al., 2023). Notably, the study found that mid-life participants who were current smokers, had a higher body mass index, or had age-related chronic diseases, tended to have a higher age acceleration in late-life. Future studies that incorporate multiple time points to model age acceleration over time are warranted. Another proteomic aging clock, developed in 2023, included 2,923 plasma proteins from 53,000 participants aged 39 to 70 (Kuo et al., 2024). This aging clock aimed to demonstrate that age acceleration estimates strongly predict all-cause mortality and disease outcomes. Interestingly, gene sets for proteins involved in epithelial mesenchymal transition, coagulation, and inflammatory response, among others, were shown to be the most influential in the functional analysis. Moreover, this clock demonstrated the strongest associations with all-cause mortality, heart failure, delirium, and cancer (Kuo et al., 2024).

Proteomic clocks may benefit from a more targeted approach where specific quantitative assessments are assessed instead of a general unbiased sieving of proteins. For example, the quantitative assessment of senescence-associated secretory phenotype that are focused on specific proteins should be explored further (Wang et al., 2024). Another example is quantitation of the klotho protein that has been associated with aging (Bhat and Ramanathan, 2024).

#### 3.2.4 Inflammatory and immune clocks

The immunological system has been implicated in many diseases of aging and have been found to be chronically elevated in the elderly (Kotas and Medzhitov, 2015). A 2021 study developed a deep learning method aging clock using the blood immunome of 1,001 individuals aged 8–96 years (Sayed et al., 2021). The resulting inflammatory clock of aging (iAge) constructed a metric that could summarize an individual’s age-related chronic inflammatory burden using the level of circulating immune proteins. During their study they also found that the chemokine CXCL9 has a key role in chronic inflammation and increases with age in blood endothelial cells (Sayed et al., 2021). Another aging clock model was developed and described in a 2023 study using single-cell RNA sequencing which revealed a ribosome-to-inflammation balance as a marker for super longevity (Zhu et al., 2023). The high ribosome level was found to contribute to a low inflammation state and the slow aging of supercentenarians. This led to the development of a single-cell level aging clock with accurate predictability of chronological age (R^2^ = 0.77) (Zhu et al., 2023). This study was notable because it suggested that aging clocks derived from single-cell lines can still provide useful information about aging. These novel studies indicate that a variety of biomarkers related to inflammation and the immunome can be utilized to determine the rate of aging. However, it is unclear how these types of clocks may be able to predict aging in relation to functional characteristics when there is little ongoing immune derangement or inflammation. Also, tissue-specific aging measures may be more difficult with these sorts of approaches.

Another inflammatory/immunological clock, termed ipAGE, expanded on the initial findings from the iAge clock by evaluating aging biomarkers in end-stage renal disease (ESRD) patients. Using 10 out of 46 inflammatory/immunological biomarkers from 76 ESRD patients and 83 healthy controls, the ipAGE was built *via* elastic net regression as a predictor of biological age and age acceleration in ESRD patients (Yusipov et al., 2022). This model demonstrated high accuracy (R^2^ = 0.79) with a MAE of 6.82 years. Significantly different biomarkers of aging in ESRD versus controls included CXCL9, CXCL10, CSF1, and IL-6, all of which have been increasingly recognized for their role in age-related diseases (Yusipov et al., 2022). This model was particularly sensitive to ESRD patients, showing a statistically significant age acceleration when compared to controls (p < 0.001). Other epigenetic clocks (DNAmPhenoAge, GrimAge) and phenotypic age also detected age acceleration in ESRD, however, there was a poor correlation between ipAGE and epigenetic clocks in ESRD, suggesting that they may capture different aspects of aging, despite both demonstrating age acceleration (Yusipov et al., 2022). Unique to ESRD pathophysiology is that it involves both immunoactivation and adaptive immune suppression. This means that ipAGE variance may reflect the heterogeneity of ESRD pathology and immune dysregulation, unlike other aging clock models. Thus, combining ipAGE with multiple clocks could provide a more comprehensive view of aging and is a potential avenue for future research.

In 2023, the development of a small immunological clock based on a limited set of immunological biomarkers, termed SImAge, was developed and trained on the same 46 immunological parameters as the previous study (Kalyakulina et al., 2023). This clock was trained using 300 healthy volunteers, and the finalized model, similar to ipAGE, used only 10 biomarkers of aging. The SImAge clock achieved a MAE of 6.94 (R^2^ = 0.939), with results comparable to other immunological clocks while using fewer input features (Kalyakulina et al., 2023). The SImAge also demonstrated significant positive age acceleration in ESRD patients, and CXCL9 was identified as the most important biomarker for age prediction across all groups. Limitations of the ipAge and SImAge model are similar, with relatively small datasets and potential for further optimization in various disease contexts.

#### 3.2.5 Neuroimaging-based clocks

Advancements in brain imaging and increases in computational analysis have enabled a better understanding of the brain as it relates to the identifiable neuroanatomical biomarkers of aging. The *BrainAge* approach attempts to predict biological age based on structural or functional markers using structural MRI data. This model has been used in numerous studies and has a high predictability for chronological age in childhood and adolescence (r = 0.93, MAE = 1.1) as well as in early to late adulthood (r = 0.92, MAE Females = 4.9, MAE Males = 5.0) (Franke and Gaser, 2019). However, BrainAGE scores varied with several chronic diseases. For example, schizophrenia patients had significantly higher BrainAGE scores by 2.6 years, patients with type 2 diabetes mellitus had a significantly increased BrainAge by 4.6 years, and health factors such as smoking, alcohol consumptions, and depression all resulted in BrainAGE differences (Franke and Gaser, 2019). Future studies and models may combine structural and functional biomarkers of brain age to refine the estimation of age and the development of various health diseases. Neuroimaging based clocks may be more useful in brain related aging predictions but its utility in predicting aging in relation to other tissues or functionality is not clear. Moreover, the development of clocks based on functional MRI, PET, and EEG may provide a more complex and accurate evaluation of brain aging.

#### 3.2.6 Microbiome-based clocks

Human microbiomes have been associated with numerous diseases, demonstrating a complex relationship with host factors to facilitate overall health. Since the discovery of age-related microbiome changes, numerous microbiome-based aging clocks have been created and these have been centered around taxonomy, biodiversity, and functional pathways.

To the best of our knowledge, there have been three microbiome aging clocks developed based on taxonomy. Two of these aging clocks were developed from gut microbiome age-related changes. In 2020, a taxonomic gut microbiome clock was developed using over 4,000 metagenomic profiles and deep learning to demonstrate associations between chronological age and microbiome composition (Galkin et al., 2020). In this study, short-chain fatty acid (SCFA) producers were the biggest influence on age estimation, followed by pathogenic bacteria (Galkin et al., 2020). Notably, a higher relative abundance of *A. muciniphila,* a SCFA producer, led to a younger predicted age while a higher abundance of *C. jejuni* shifted led to a higher predicted age compared to chronological age. Another taxonomic gut microbiome clock developed from 4,434 fecal samples in 2020 demonstrated similar results (Huang et al., 2020). Interestingly, SCFA producers including bacteria from the genera *Bifidobacterium* and *Blautia* or symbiotic bacteria from the families *Lachnospiraceae*, *Ruminococcaceae,* and *Clostridiaceae* were found to be most influential (Huang et al., 2020). The last aging clock was developed on skin microbiome age-related taxonomic changes. This aging clock utilized 16S rRNA sequencing data from nearly 2,000 skin samples (Huang et al., 2020). The most influential taxa in this aging model included *Mycoplasma*, *Enterobacteriaceae*, and *Pasteurellaceae*, which were all negatively correlated with age (Huang et al., 2020).

The use of 16S rRNA sequencing, instead of a whole-genome sequencing approach, prevents a direct measurement of the functionality of the gut. Whole genome sequencing allows for identification of the presence of a gene but this approach still requires validation against other gold standard measurements. Overall, while microbiome based aging clocks have potential, there is still much work and research to be done to optimize gene presence and better functional predictions. Apart from the sequencing issues, future research should consider objective measurements for the baseline skin pigmentation, as there are differences associated with skin aging based on baseline pigmentation level or other objective measures of skin type (Vashi et al., 2016).

Microbiome biodiversity has also been implicated in aging. Thus, two biodiversity aging clocks have resulted. In 2020, 1,649 publicly available 16S rRNA sequencing data sets was utilized to develop a biodiversity-based aging clock model (Sala et al., 2020). Notably, there was a decrease in microbial biodiversity with increasing age (Sala et al., 2020). Another 2021 study investigated gut microbiome and phenotypic data from over 9,000 individuals demonstrated that the β-diversity of gut microbiomes become more unique with age, and that these differences may related to metabolic changes (Wilmanski et al., 2021).

One step further than taxonomic and biodiversity-based aging clocks are functional clocks, which consider both composition and functionality. These aging clocks utilize metagenomic sequencing to characterize metabolic pathways and enzymes along with taxonomy. One 2022 study performed metagenomic analysis of 4,478 fecal samples to create an aging clock model (Chen et al., 2022). Combining the resulting taxonomic and functional models led to the development of a refined functional aging clock model. Among all taxonomic and functional factors, the most predictive included acetyl-CoA biosynthesis, nicotinate degradation, and *Finegoldia magna*. There were several species that increased with age including *F. magna*, *Bifidobacterium dentium*, and *Clostridium clostridioforme*. There were also a few that decreased with age including *Prevotella copri* and *Burkholderialse* bacterium (Chen et al., 2022). Another 2024 aging clock utilized 90,303 fecal samples to similarly create a functional aging clock by correlating the gut bacteria with transcriptome based measurements in the gut (Gopu et al., 2024). Interestingly, the species that were most predictive included *Ruminococcaceae*, *Bifidobacteriaceae*, *Lachnospiraceae*, and *Clostridiaceae* families and these findings agree with a previously mentioned gut microbiome aging model (Galkin et al., 2020; Gopu et al., 2024). The strength of this study is that the gene expression was directly measured to assess for functional profiling rather than extrapolating present bacteria with function. Additionally, there were several metabolic pathways that were associated with aging including vitamin B12 biosynthesis, amino acid metabolism, and SCFA production (Gopu et al., 2024).

#### 3.2.7 Glycomic analysis based clocks

Glycomic age prediction analyzes drift across immunoglobulin G (IgG) glycosylation, where changes during aging have been observed through inflammatory and autoimmune processes (Palmer, 2022; d’Adda di Fagagna et al., 2003). By measuring these glycan signatures, an aging clock can be constructed. For example, one of the causes of skin aging is advanced glycation end products (AGEs). Glucose autoxidation and the covalent attachment of glucose molecules to proteins result in the formation of AGEs and oxidative stress (Kim et al., 2017). AGEs accumulate in the skin as it ages, negatively affecting the extracellular matrix causing a loss of elasticity and inflammation (Pageon et al., 2014). Hemoglobin A1c should be incorporated as part of any clock that utilizes a glycomic analysis based approach based on previous modeling work in regards to aging (Bhat and Ramanathan, 2024). To the best of our knowledge, glycomic analysis is an emerging topic and more research is warranted to develop an aging clock based on the glycome.

#### 3.2.8 Visual facial skin aging clock

DNAm patterns are a characteristic of aged skin, and its visible signs of aging are an opportunity to study the aging process. One such study developed a facial aging clock, VisAgeX, that predicts the skin aging phenotype including wrinkle grade, visual facial age, and visual age progression. The clock used gene expression and methylation data to better understand the pathways of skin aging. The authors demonstrated that visual age is a reliable measure of age progression as chronological age is strongly correlated with observed facial age (R = 0.91, p = 2.20E-16) (Bienkowska et al., 2023). Another potential clock is the “facial aging clock” which was proposed by Wang, et al. This clock relies on deep learning based facial image technologies and may be less invasive and expensive than the DNA methylation clock. They also purport to provide an objective identification of disease risk and progression, and an evaluation of ongoing therapies. (Wang Y. et al., 2023). A limitation with visual based skin aging clocks is that it does not consider the skin biophysical features of the face such as transepidermal water loss, hydration, or elasticity measurements. Another limitation is that facial aging clocks have largely been trained on non-diseased facial images without clinician validation. Also, facial cosmetic procedures or surgeries may serve as a confounding factor when utilizing visual assessment measures that rely on visual appearance alone and may not reflect true aging of the entire body beyond the face. Future research studies should incorporate more objective and biophysical measures of the skin and consider the use of biopsies to correlate against histological changes in the skin.

#### 3.2.9 Echocardiography based aging clock

A novel neural network model trained on 31 echocardiographic parameters was developed in 2024, trained on a dataset of 10,698 echocardiographic examinations from 9,901 individuals aged 18–96 years (Kobelyatskaya et al., 2024). The model, named EchoAGE, predicts heart biological age, and demonstrated high accuracy in predicting chronological age, with a MAE of 5.76 years (R^2^ = 0.91). Key echocardiographic parameters contributing to aging included left atrial volume index, E/e’ ratio, and left ventricular mass index. Notably, EchoAGE demonstrated the ability to detect accelerated cardiac aging in patients with various cardiovascular conditions (Kobelyatskaya et al., 2024). For example, heart failure patients had a predicted heart age 8.9 years older than their chronological age, hypertensive patients demonstrated a 3.2-year acceleration in heart age, and coronary artery disease patients had a 6.5-year acceleration. Interestingly, the model also identified decelerated aging in athletes, with a predicted heart age 5.7-year younger than their chronological age (Kobelyatskaya et al., 2024). Future research may consider focusing on longitudinal studies to validate the prognostic value of EchoAGE and its applications in guiding prevention strategies and therapeutic interventions for cardiovascular disease.

### 3.3 Factors that influence aging clocks

There are several factors that may influence aging clock predictions including comorbid disease and health conditions, lifestyle factors such as exercise and diet and psychosocial factors. Aging clocks use biomarkers for disease to predict biological age. A complex system of risk factors is included for analysis in the algorithms and calibration processes of aging clocks. These may include obesity, diabetes, cardiovascular disease, neurodegenerative diseases (Margiotti et al., 2023). Thus, confounding diseases and health conditions may interfere with aging clock predictions.

#### 3.3.1 Lifestyle, diet, and nutrition

There is strong evidence that lifestyle factors affect health outcomes. Diet and nutrition, alcohol consumption, physical activity, and educational attainment have been shown to affect health and aging, and it has been shown that these factors directly influence aging on a molecular level (Quach et al., 2017). One study showed a small but significant reduction in DNAmAge in individuals consuming a lean meat, fish and plant-based diet. Compared to a control group, those participating in the treatment group scored an average of 3.23 years younger at the end of the 8-week program using the Horvath DNAmAge clock genome analysis (Fitzgerald et al., 2022). Another study included diet, lifestyle guidance, stress reduction, and sleep optimization. Compared to the control group, participants in the treatment group scored an average 3.23 years younger at the end of the 8-week program according to the Horvath DNAmAge clock (Fitzgerald et al., 2021) A study of a year’s duration reports that a Mediterranean-like nutritional intervention can promote epigenetic rejuvenation in the elderly (Gensous et al., 2020). Another trial demonstrated that long-term caloric restriction led to a significant 2%–3% reduction in the pace of aging at 12 (*d* = −0.29) and 24 months (*d* = −0.25), as measured by the DunedinPACE aging clock and interpreted utilizing standardized differences between means (Cohen’s *d*). However, measures of aging rate by other DNAm clocks including PhenoAge and GrimAge were unaffected (Waziry et al., 2023).

#### 3.3.2 Psychosocial

Psychosocial factors may substantially influence aging and have a direct effect on the aging clock. One study found that factors such as feeling unhappy or lonely add up to 1.65 years to one’s biological age (Galkin et al., 2022). A study on baboons linked social stress caused by competition to reversible aging acceleration (Phillips et al., 1988). In a human clinical study, it was observed that accumulated stress can be responsible for up to 3.6 years of extra biological aging (Zannas et al., 2015). Newer research has demonstrated that trauma increases epigenetic aging, and that some psychosocial factors can protect from biological aging, and some factors can reverse this aging process (Mehta et al., 2022). In terms of biomarkers for stress, future research should consider incorporation of cortisol and cortisol related markers as they influence inflammation and immune system function which may influence biological aging (Munoz-Durango et al., 2015; Ravi et al., 2021).

### 3.4 Challenges and limitations

One limitation of the current aging clocks is that there is some variability among clocks that are more useful in estimating chronological age, all-cause mortality, visual age, and other measures of aging versus biological age. Thus, not all aging clocks may be predictive of aging rate. Moreover, it is important to consider which of the several clocks are being used as well as their strengths and weaknesses, the tissues sampled, and the number of CpGs present. With increasing training sample size, improved measurement of chronological age is expected (Bell et al., 2019). During a study on aging in Hutchinson Gilford Progeria Syndrome, it was found that epigenetic age changes in fibroblasts were detected with the Hannum skin and blood clock whereas the pan-tissue clock by Horvath did not detect the age changes in this case. As such, one of the challenges is to choose the correct type of testing for the type of study or research (Horvath et al., 2018).

Another major challenge is to determine the individual contributing factors, how they interact and their relative contributions to aging, with the final goal of identifying pharmaceutical targets to improve human health during aging, with minimal side effects (Lopez-Otin et al., 2013). Also, there is population variation in the rate at which people age. A further caution in the utilization of aging clocks is that much of our understanding of the biology of aging comes from studies on model organisms, such as mouse or baboon research data. Additional research involving human subjects is currently in progress to acquire detailed understanding of the impact of the aging clock and potential interventions that can slow or reverse the aging process.

Skin based aging clocks need to utilize skin biophysical features in addition to visual or photography-based analysis. Aging is not just a visual phenomenon on the face as biophysical shifts in elasticity and hydration likely contribute to the overall age of the skin. Pigment and erythema should be captured with objective and validated devices to measure the erythema intensity and the true pigment measure rather than crude placement into the Fitzpatrick skin types.

Ethical considerations surrounding biological age prediction include risks to privacy, potential misuse of sensitive data, and exacerbation of health inequities if aging clocks are not designed using diverse populations. Moreover, there are potential psychological impacts of revealing biological age, especially in scenarios where the chronological age differs significantly. This highlights the need for ongoing continued research to further refine how biological age calculations are performed. Lastly, the commercialization of aging products without rigorous validation risks consequences to consumers and may create inequitable access to technologies. Addressing these concerns is essential to ensure ethical usage of biological age predictions.

There are also limitations due to standardization of samples. Many genetic and epigenetic data and analyses are strongly biased toward populations of European ancestry and other populations are grossly under-represented. This is a similar limitation to aging clocks based on skin samples, given that diversity in skin types may not be accounted for. Fitzpatrick skin type is also subjective in nature, and methodology focused on objective measurement of skin type such as individual typology angle measures or melanin content should be utilized (Osto et al., 2022). Moreover, technical noise in epigenetic data accounts for deviations of 3–9 years among 6 major epigenetic clocks, which can drastically affect the reliability of aging clocks (Higgins-Chen et al., 2022). One solution to this limitation has become the use of additional statistical tests to correct for unwanted variation (Higgins-Chen et al., 2022; Perrier et al., 2018). Among these correcting techniques include principal component analysis, which extracts shared age-related changes without picking up variations from individual noncontributory CpGs, surrogate variables analysis, ComBat, and linear regression models (Higgins-Chen et al., 2022; Perrier et al., 2018). Increased technical noise and reduced model accuracy are some examples of the impact of batch effect on epigenetic data, otherwise defined as non-biological variations in data that arise from differences in experimental conditions or technical processes. Additional sources of batch effect in epigenetic data also include differences in equipment, variations in reagents, lab personnel, or environmental conditions, as well as timing, platform-specific differences, and data handling or processing. Strategies to mitigate batch effect may include randomization, processing all samples on the same platform whenever possible, normalization methods as mentioned previously, quality control, and cross-batch validation. Moreover, the breadth of currently available aging clocks is quite expansive. Our search strategy considered numerous factors including epigenetic changes, the proteome, microbiome, telomeres, and more. However, given the breadth of available studies, the search strategy may have excluded aging clock models within more niche realms, such as aging clocks based on specific diseased populations.

### 3.5 Clinical applications and future directions

Continued research into the aging clock may allow pre-emptive targeted health-promoting interventions in a population based, or in a personalized and disease-specific fashion. The future of this science appears to be in testing interventions that attempt to slow or reverse the aging process. The mechanisms of aging are a complex interconnected web of genetic and epigenetic factors. Further study of aging clocks will facilitate future interventions for improving human health and longevity. These might include epigenetic drugs, stem cell-based strategies, clearance of senescent cells, DR, IIS, and mTOR inhibition, AMPK and sirtuins activation (Lopez-Otin et al., 2023). The future may lie in new technologies and deep learning applications. Few sequencing-based studies of chronological aging clocks have investigated regions beyond the CpGs profiled using array-based techniques. It may not be cost effective to use deep sequencing-based studies especially with whole-genome base-resolution techniques where Horvath’s pan-tissue clock exploits the 27k array and is highly accurate in predicting chronological age (Bell et al., 2019). However, research into epigenetic factors which slow the aging clock will continue to expand our understanding and lead to better measures of biological age as it pertains to functional status. For example, studies that focused on reversing the DNA methylation profile brought to light a class of drugs called epidrugs, which lead to changes in gene expression. Some DNA methyltransferase inhibitors (DNMTi) have been approved by the FDA and are in use in oncological diseases (de Oliveira et al., 2020).

Aging clocks also provide clinicians with a means of tracking disease progression. The measurement of biological age before and after treatment can give insight into the systemic efficacy of the drug. Not only does this allow for comparability between conditions and treatments, but also gives us proof behind the systemic burden of a condition. For example, early research has demonstrated that biological age is increased in children with atopic dermatitis (Jeremian et al., 2024), suggesting that this is not only a skin condition but a systemic inflammatory condition. There also continues to be developments of novel aging clocks. Future research using new technologies and deep learning models may produce an entirely new aging clock, or a more robust and accurate model using a combination of approaches and biomarkers.

## 4 Conclusion

We summarize the current published research in aging clocks, factors that affect their predictability, their limitations, clinical applications, and future directions. Currently, there are numerous established aging clocks based on epigenetic changes, proteomic changes, inflammatory and immune pathways, neuroimaging, microbiome-associated changes, among other novel aging clocks models. Interindividual variability in confounding diseases, lifestyle factors, and psychosocial factors may all influence the predictability of aging clock models. Further, the variety of sample tissues, which age at different rates makes a constant aging rate difficult to determine. The use of AI and deep neural networks will continue to accelerate research into biomarker discovery and predictions in aging. Aging clock research will continue to shed light on the physiological processes of aging and develop therapeutic strategies and interventions with the goal of decelerating aging and improving the prevalence of chronic diseases in an aging population.
