# Supplementary material for: Critical review of aging clocks and factors that may influence the pace of aging
Source: Front Aging. 2024 Dec 13;5:1487260. doi: 10.3389/fragi.2024.1487260 (PMC11671503; doi:10.3389/fragi.2024.1487260)
Supplement: Supplementary file 1 [file Table1.docx]

Supplementary Material

**Table 1.** Summary of Aging Clocks and Predictability Against Chronological Age

| **Author (Year)** | **Method** | **Most significant CPG genes, proteins, biomarkers, etc.** | **Sample** | **R^2^** | **Mean Absolute Error (Years)** |
| --- | --- | --- | --- | --- | --- |
| Hannum et al. (2013) (7) | Epigenetic: DNA Methylation | 71 methylation markers notably including cg27193080, cg18404041, cg05652533, and cg27367526 | Whole blood | 0.96 | 3.9 |
| Horvath (2013) (8) | Epigenetic: DNA Methylation | 353 CpGs, 193 are positively correlated with age, 160 are negatively correlated with age | Multiple tissues/cells | 0.96 | 3.6 |
| Levine et al. (2018) (10) | Epigenetic: DNA Methylation + Clinical Outcomes | 513 CpGs notably including cg05442902, cg06493994, cg09809672, cg19722847, and cg22736354 | Whole blood | 0.68 | * |
| Lu et al. (2019) (11) | DNA Methylation-based plasma proteins + pack/years of smoking | ADM, B2M, Cystatin C, GDF-15, Leptin, PAI-1, and TIMP-1 | Whole blood | * | * |
| Weidner et al. (2014) (12) | 3 Age-related CpGs | ITGA2B, ASPA and PDE4C | Whole blood | 0.98 | MAD: 3.34 |
| Jenkins et al. (2018) (13) | Epigenetic: DNA Methylation | 51 regions notably including ADAMTS8, ARC, FOXK1, PAX2, etc. | Sperm cells | 0.89 | 2.04 |
| Belsky et al. (2020) (14) | Epigenetic: DNA Methylation + biomarkers | 46 CpGs notably including genes including ACTBP11, PPARG, XXYLT1, MIR518A2, etc. and 19 biomarkers (Table 1) | Whole blood | * | * |
| Belsky et al. (2022) (15) | Epigenetic: DNA Methylation + biomarkers | 46 CpGs notably including genes including ACTBP11, PPARG, XXYLT1, MIR518A2, etc. and 19 biomarkers (Table 1) | Whole blood | * | * |
| Galkin et al. (2021) (16) | Epigenetic: DNA Methylation | 1000 CpGs, notably enriched CpG sites were involved in tissue development, organ morphogenesis, neural function, etc. | Whole blood | 0.93 | 3.80 |
| McGreevy et al. (2023) (17) | DNAmGrimAge + DNAm biomarkers for fitness | 627 CpGs, notably including genes ZNRD1, HLA-G, MHC, and tapasin binding | Whole blood | 0.77 | MAD: 2.3 to 4.9 |
| Tanaka et al. (2018) (24) | Proteomic | 217 proteins notably including GDF15, PTN, ADAMTS5, FSH, SOST, CHRDL1, NPPB, FBLN3, MMP12, and CTSV | Plasma | 0.94 | MAD: 5.7 |
| Sathyan et al. (2020) (25) | Proteomic | 754 proteins, notably including PTN, WISP-2, CHRDL1, TAGL, and RSPO1 | Plasma | 0.79 | * |
| Lehallier et al. (2020) (26) | Proteomic | 529 proteins, notably including CGA, FSHB, SOST, GDF15, MLN, RET, and PTN | Plasma | 0.96 | 2.44 |
| Wang et al. (2023) (27) | Proteomic | 788 aptamers for mid-life and 135 aptamers for late-life. Notable: PTN, ADAMTS5, MMP12, and CDON | Plasma | Mid-life  0.80  Late-life  0.71 | Mid-life  2.19  Late-life  2.36 |
| Kuo et al. (2023) (23) | Proteomic | Genes for epithelial mesenchymal transition, coagulation, and inflammatory response | Plasma | 0.77 | * |
| Sayed et al. (2021) (31) | Immunome | Positive: CXCL9, EOTAXIN, Mip-1α, LEPTIN, IL-1β, IL-5, IFN- α, IL-4  Negative: TRAIL, IFN- γ, CXCL1, IL-2, TGF-α, PAI-1, and LIF | Whole blood | * | * |
| Zhu et al. (2023) (32) | Single-cell RNA sequence from super centenarians | Delayed age-dependent decrease in CD8+ naïve T cells, Naïve B cells, and Memory B cells. Delayed age-dependent increase in CD4+ Tm | Human Peripheral Blood Mononuclear Cell | 0.77 | MAD: 8.36 |
| Yusipov et al. (2022) (33) | Immunome | 38 biomarkers including CXCL9, CXCL10, IL-15, and TNFRII | Plasma Biomarkers | 0.79 | 6.82 |
| Kalyakulina et al. (2023) (34) | Immunome | 10 selected biomarkers including: IL-6, CSF-1, PDGFA, CXCL10, CXCL9, CCL22, PDGFB, and VEGFA | Plasma Biomarkers | 0.94 | 6.94 |
| Franke et al. (2019) (35) | Neuroimaging | N/A | Structural MRI data | Adolescents 0.93  Adults  0.92 | Adolescents  1.1  Adults  F: 4.9  M: 5.0 |
| Galkin et al. (2020) (36) | Microbiome: Taxonomic | *Bifidobacterium spp*., *A. muciniphila*, and *Bacteroides spp.,* *E. coli* and *C. jejuni* | Gut microbiome | 0.20 | 10.6 |
| Huang et al. (2020) (37) | Microbiome: Taxonomic | *Bifidobacterium, Blautia, Lachnospiraceae*, *Ruminococcaceae,* and *Clostridiaceae* | Gut microbiome | 0.17 | 11.5 |
| Huang et al. (2020) (37) | Microbiome: Taxonomic | *Mycoplasma*, *Enterobacteriaceae*, and *Pasteurellaceae* | Skin microbiome | 0.74 | 3.8 |
| Sala et al. (2020) (39) | Microbiome: Biodiversity | Decreased biodiversity with age | Gut microbiome | * | * |
| Wilmanski et al. (2021) (40) | Microbiome: Biodiversity | β-diversity of gut microbiomes become more unique with age | Gut microbiome | * | * |
| Chen et al. (2022) (41) | Microbiome: Functional | Acetyl-CoA biosynthesis, nicotinate degradation, and *Finegoldia magna*. | Gut microbiome | 0.6 | 8.3 |
| Gopu et al. (2024) (42) | Microbiome: Functional | *Ruminococcaceae*, *Bifidobacteriaceae*, *Lachnospiraceae*, and *Clostridiaceae;* Vitamin B12 biosynthesis, amino acid metabolism, and SCFA production | Gut microbiome | 0.42 | 9.5 |
| Bienkowska et al. (2024) (45) | Visual Skin Aging + Epigenetic: DNA Methylation | Genes related to hypoxia, estrogen responses, IL2 STAT5 signaling. | Portrait images and gene expression data | 0.91 | 5.76 |
| Wang et al. (2023) (46) | Visual Skin Aging | N/A | Portrait images | * | * |
| Kobelyatskaya et al. (2024) (47) | Echocardiography | RWT, IVS, LVPW, EA, and LVCO | Echocardiography | 0.9 | 5.76 |

^*These studies did not perform either error descriptions or linear correlation analyses.^

^MAD, mean absolute deviation (years); F, female; M, male; ADM, adrenomedullin; B2M, beta-2-microglobulim; GDF-15, growth differentiation factor 15; PAI-1, plasminogen activator inhibitor 1; TIMP-1, tissue inhibitor metalloproteinases 1; ITGA2B, integrin alpha 2b; ASPA, aspartoacylase; PDE4C, phosphodiesterase 4c; ADAMTS8, ADAM metallopeptidase with thrombospondin type 1 motif 8; ARC, activity regulated cytoskeleton associated protein; FOXK1, forkhead box K1; ACTBP11, actin beta pseudogene 11; PPARG, peroxisome proliferator activated receptor gamma; XXYLT1, xyloside xylosyltransferase 1; MIR518A2, microRNA 518A2; ZNRD1, zinc ribbon domain-containing 1; HLA-G, histocompatibility locus antigen G; MHC, major histocompatibility complex; GDF15, growth differentiating factor 15; PTN, pleiotrophin; ADAMTS5, ADAM metallopeptidase with thrombospondin type 1 motif 5; FSH, follicle stimulating hormone; SOST, sclerostin; CHRDL1, chordin‐like protein 1; NPPB, natriuretic peptide B; FBLN3, EGF‐containing fibulin‐like extracellular matrix protein 1; MMP12, matrix metallopeptidase 12; CTSV, cathepsin V; WISP-2, WNT1‐inducible‐signaling pathway protein 2; TAGL, transgelin; RSPO1, R‐spondin‐1; CGA, glycoprotein hormone alpha polypeptide; FSHB, follicle stimulating hormone subunit beta; MLN, motilin; CXCL, chemokine ligand; Mip-1α, macrophage inflammatory protein 1 alpha; IL, interleukin; IFN, interferon; TRAIL, TNF-related apoptosis-inducing ligand; TGF, transforming growth factor; PAI-1, plasminogen activator inhibitor 1; LIF, leukemia inhibitory factor; TNFRII, tumor necrosis factor receptor II; CSF, colony-stimulating factor; PDGFA, platelet-derived growth factor subunit A; CCL, C-C motif chemokine ligand; PDGFB, platelet-derived growth factor subunit B; VEGFA, vascular endothelial growth factor A; LVCO, cardiac output (L/minute); EA, E/A ratio of maximum flow rates in the first and second phases; RWT, relative wall thickness; IVS, thickness of the interventricular septum (cm); LVPW, thickness of the posterior left ventricular wall (cm).^
